# Vaccines for cancer interception in familial adenomatous polyposis

**DOI:** 10.3389/fimmu.2025.1525157

**Published:** 2025-01-29

**Authors:** David E. Johnson, Mary L. Disis

**Affiliations:** ^1^ Ben Towne Center for Childhood Cancer Research, Seattle Children’s Research Institute, Seattle, WA, United States; ^2^ UW Medicine Cancer Vaccine Institute, University of Washington, Seattle, WA, United States

**Keywords:** vaccine, familial adenomatous polyposis (FAP), chemoprevention, cancer interception, T-cells

## Abstract

Familial adenomatous polyposis (FAP) is an inherited autosomal dominant disorder caused by germline mutations in the adenomatous polyposis coli (APC) gene. FAP is associated with the development of hundreds of adenomas in the small and large intestines of individuals starting in the teenage years with a near 100% risk of developing colorectal cancer by adulthood. Eventually polyps develop throughout the gastrointestinal tract. Chemoprevention approaches have been somewhat successful in reducing polyp burden, but have not reduced the risk of the development of colorectal cancer or other cancers. The lack of efficacy of more standard drug approaches may be due to limited exposure to the agent only to specific periods while the drug is being metabolized, limited drug penetrance in the colon, and patient adherence to daily dosing and drug side effects. The success of immune therapy for the treatment of invasive cancer has led to research focused on the use of immune based approaches for polyp control in FAP, specifically polyp directed vaccines. Vaccines targeting antigens expressed in FAP lesions may be a superior method to control polyp burden and prevent disease progression as compared to classic chemoprevention drugs. A limited number of vaccines can be administered over a short period of time to generate a lasting immune response. Appropriately primed antigen specific T-cells can traffic to any site in the body where antigen is expressed, recognize, and eliminate the antigen expressing cell. Immunologic memory will allow the immune response to persist and the specificity of the immune response will limit toxicity to the targeted polyp. This review will examine the current state of vaccines directed against FAP lesions and highlight the challenges and opportunities of translating vaccines for cancer interception in FAP to the clinic.

## Introduction

Familial adenomatous polyposis (FAP) is an inherited autosomal dominant disorder caused by germline mutations in the adenomatous polyposis coli (APC) gene. The disorder is characterized by the development of hundreds of adenomas in the rectum and colon of individuals starting in the teenage years with a near 100% risk of developing colorectal cancer (CRC) by adulthood. FAP is the most common adenomatous polyposis syndrome and is responsible for approximately 1% of all CRC diagnoses. Although FAP is largely an inherited disease, 25% of cases can occur *de novo* in patients with no family history (1). There is much morbidity associated with the disease. The treatment of FAP is based on frequent cancer surveillance with multiple endoscopies during the patient’s lifetime until surgery is warranted. A high polyp burden in the intestine is associated with significant symptoms including rectal bleeding, abdominal pain, anemia, and diarrhea. Unfortunately, FAP is present throughout the entire intestine and removal of the colon will not entirely eliminate cancer risk. Chemoprevention approaches have been somewhat successful in reducing polyp burden, but have not reduced the risk of the development of colorectal cancer or other intestinal cancers.

Vaccines targeting antigens expressed in FAP lesions have been explored as a method to control polyp burden and prevent disease progression. Vaccines have several theoretical advantages as a prevention approach in FAP as compared to classic chemoprevention drugs. A limited number of vaccines can be administered over a short period of time to generate a lasting immune response. Appropriately primed antigen specific T-cells can traffic to any site in the body where antigen is expressed, recognize, and eliminate the antigen expressing cell. Immunologic memory will allow the immune response to persist and the specificity of the immune response will potentially limit any toxicity to the targeted polyp. This review will examine the current state of vaccines directed against FAP lesions and highlight the challenges and opportunities of translating vaccines for cancer interception in FAP to the clinic.

## Treatment of FAP

The foundation of the management of FAP is aggressive endoscopic screening starting between 10-15 years of age with annual colonoscopy and polypectomy when needed (2). Due to the later age of onset of upper intestinal polyps, upper endoscopy is initiated annually at a later age; about 20-25 years (2). A proctocolectomy or total colectomy is indicated when polyps become large >10mm, high grade dysplasia or colorectal cancer develops, or endoscopic surveillance is not possible (3). Unfortunately, there is an ongoing risk of the development of both intestinal and extraintestinal cancers (e.g. gastric, thyroid) even after surgical treatment. Because of the inability to totally remove pre-malignant polyps, there has been an effort to identify a chemoprevention approach for FAP. A systemic therapy might allow a delay in polyp formation, allow for less extensive surgeries to be performed later in life, and potentially decrease patient morbidity. Moreover, a systemic drug-based approach to the disease could prevent progression of dysplasia to invasive cancer in multiple tissues simultaneously (4–6).

To date, several agents have been identified that can reduce the polyp burden to a certain extent, but none of these have been shown to reduce the incidence of cancer (6). Given COX2 is highly expressed in polyps in FAP, the most commonly used chemotherapy to reduce polyp burden are cyclooxygenase inhibitors such as celecoxib and sulindac (7). Indeed, in a randomized clinical trial, 6 months of celecoxib at a 400-mcg dose could reduce polyp burden by 30% (8). Several studies have used celecoxib, or other cyclooxygenase inhibitors, as the backbone of combination therapy with additive effects. One double blinded controlled trial randomized 92 patients with FAP to sulindac, a COX-1 and COX-2 inhibitor, and erlotinib, an EGFR inhibitor vs. placebo (9). The combination treated group had a 37.9% decrease in duodenal polyp burden from baseline as compared to a 30.6% increase observed in the placebo treated group. A limitation to the study was the toxicity seen with the treatment; 87% of treated patients experienced acneiform rash due to the erlotinib and adverse events were more common in the combination therapy treated group. Another trial tested the combination of celecoxib vs. celecoxib plus diflouromethylornithine in 112 randomized patients. The primary endpoint of the study was a decrease in adenoma count, but after treatment, there was no difference between the two groups (10). In addition to cyclooxygenase inhibitors, a variety of compounds have been tested as chemoprevention drugs in FAP such as mTOR inhibitors, natural products, and antibiotics to name a few, but none have shown the ability to prevent the development of CRC (5). There are currently many agents under investigation in both pre-clinical models as well as clinical trials for the chemoprevention of polyps in FAP with the hope these drugs will eventually prevent colorectal cancer. A list of active chemoprevention clinical trials is shown in **Table 1**.

**Table 1:** Clinical trials for polyp/cancer prevention in familial adenomatous polyposis (FAP).

Active studies that are currently recruiting FAP patients		
NCT Number	Intervention	Primary Objective
NCT06545526	Combination of celecoxib and metformin	Percent change in the number and size of polyps in the colon and/or duodenum
NCT06578637	β-hydroxybutyrate supplementation	Safety and tolerability
NCT05223036	Obeticholic acid	Reduction of duodenal polyp burden
Active studies that are not yet recruiting FAP patients		
NCT Number	Intervention	Primary Objective
NCT06557733	TPST-1495 (dual antagonist of prostaglandin E2 receptor)	Reduction of duodenal polyp burden
NCT06308445	Rapamycin	Safety and tolerability
NCT06641310	Exercise therapy	Compliance
NCT05630794	ONC20 (Akt/Erk inhibitor)	Optimal cancer prevention dose based on toxicity and biomarker endpoints

Data as of 12/12/2024.

Vaccines are a novel approach to cancer interception and offer many advantages over drug therapy. A limited number of immunizations can be given over a short period of time to achieve the desired immune response. Maintenance of that immune response can be maintained with periodic booster immunizations. If immunologic memory develops, antigen specific T-cells will persist in the body for years, ready to be deployed when aberrant cells/antigens are detected. Moreover, appropriately primed T-cells can migrate to any tissues in the body that are expressing antigen, including the colon. Further, antigen specific T-cells will continue to respond to and kill the target until no antigen expressing cells are remaining. These characteristics, which can lead to potential complete polyp control, have led investigators to explore the development of vaccines aimed to eliminate or control polyp formation in FAP.

## Vaccines directed against polyps in FAP

The APC^Min^ mouse model has facilitated the development and testing of vaccines directed against polyps associated with FAP. C57BL/6j(B6) ^Min+^ mice develop over 50 tumors throughout both the upper and lower intestinal tract. Most tumors are benign adenomas and the mice will die from the side effects of the disease, anemia, bleeding, and intestinal obstruction, rather than frank malignancy (11). This mouse model shares several similarities with human FAP; multiple colon and small intestinal adenomas, development of desmoid tumors, and similar response to therapies used to treat the disease (11, 12). A meta-analysis of studies evaluating the effect of aspirin therapy on the inhibition of polyps in patients with FAP and the APC^Min^ demonstrated that the anti-tumor efficacy observed in the mouse model predicted the clinical effect observed in humans (12).

The intestinal microenvironment in FAP is immunologically active with a balance between gut tolerance and inflammation being maintained by the ratio of T-regulatory (T-reg) to CD4+ T-helper 17 (Th17) cells. Th17 cells, which secrete IL-17A, are overabundant in most inflammatory gastrointestinal disease states such as ulcerative colitis, Crohn’s disease, and inflammatory bowel disease where these cells are associated with the generation of fibrosis and the development of inflamed lesions that progress to malignancy (13). In the APC^Min^, Th17 cells have been shown to support the development of tumors (14). Investigators created an IL-17A deficient APC^Min^ mouse (IL-17A KO-APC^Min/+^) and demonstrated that animals depleted of IL-17A had a significant decrease, over 80%, in the number of tumors in both the small and large intestine as compared to mice with intact expression of IL-17A (14). Further studies have shown that APC^Min^ mice, as compared to wild type have decreased levels of IFN-gamma/IL-17 double positive CD4 T-cells in the mesenteric lymph nodes and Peyers patches, have reduced levels of CD8 cells with reduced production of IFN-gamma and granzyme B which are needed for direct cell killing of cancer (15). These defects significantly impact the ability to prevent the development of intestinal dysplasia and adversely impact cancer immune surveillance. One way to replace IFN-gamma secreting T-cells is via active immunization and one of the first studies using a vaccine to inhibit polyp formation in the APC^min^ was published over 2 decades ago (16). Bone marrow derived dendritic cells (DC) were loaded with syngeneic intestinal tumor cells derived from the transgenic mouse using polyethylene glycol treatment and administered as a vaccine. IL-12 was then delivered intraperitoneally concurrent with vaccinations. Significant polyp inhibition was observed in immunized animals as compared with controls and was associated with the development of high IgG1 antibody titers against tumor cells. The anti-polyp response was found to be mediated by CD4 T-cells in depletion studies performed in vaccinated and control mice (16). As vaccine technologies have evolved over the ensuing years, antigen specific approaches have become the focus of vaccines targeting polyposis syndromes. This focus is most likely due to several factors including (1) the ease of standardization of antigen specific vs. cell-based vaccine manufacture, (2) the recent ability to better define immunogenic proteins in polyps associated with FAP (17), and (3) new vaccine technologies that allow more rapid and inexpensive manufacture of antigen-specific vaccines (18).

Antigen specific vaccines targeting FAP are in development and directed against two broad classes of antigens (1) specific mutated proteins associated with a particular APC mutation or (2) aberrantly expressed non-mutated tumor associated proteins (**Table 2**). Vaccines directed against mutations that arise in cancer are appealing as these “neoantigens” are new to the immune system and there is little chance of developing tolerance. In addition, these mutated antigens are inherently more immunogenic than non-mutated proteins. In patients with a variety of invasive malignancies, immunizing with vaccines encompassing multiple mutations expressed by their cancers encoded in short epitopes can elicit a potent anti-tumor response. This principle was demonstrated in one study, in which patients with pancreatic cancer were immunized with up to 20 neoantigens. Results showed that patients whose T-cell response persisted after immunization enjoyed a longer time to disease recurrence as compared to those patients whose immunity waned (19). Although FAP is caused by a germline APC mutation, the polyps that arise in patients have a lower mutation rate, about 5 mutations/megabase as compared to highly mutated tumors which have greater than 10 mutations/megabase (20). Generating vaccines encoding multiple mutated sequences, as was done in the pancreatic cancer study, simply could not be achieved in for FAP lesions. The polyps would require a greater tumor mutational burden to have a chance at identifying enough neoepitopes with the potential to bind to the patients’ MHC (21). All patients with FAP, however, carry a germline mutation which can encode a mutated protein and investigators have shown that a “neoepitope” derived from such a germline APC mutated protein can be identified (22) (**Table 2**). Researchers identified a novel mutation in the FAP gene in 10 of 26 family members, 6 of which were impacted by polyposis syndrome and 4 individuals who were polyp free. Bioinformatics approaches were utilized to identify peptides which harbored the novel mutation and that had a high likelihood of interaction with the Class I MHC identified for the 10 individuals. Peripheral blood lymphocytes were interrogated for interaction with the proposed neoepitopes. Cytotoxic T-cell (CTL) responses to one neoepitope could be detected in volunteer donors or in family members who were not APC gene carriers, but not in the individuals who did carry the mutation suggesting that they had been tolerized to the mutation. The ability of the neoepitope to be recognized by a non-mutation carrier provides proof of principle that vaccines directed against APC germline mutations can be developed. Vaccine methodologies employed for non-mutated antigens, however, designed to circumvent tolerance would potentially need to be used with APC mutation-based vaccines when immunizing patients harboring those germline mutations as they could already be tolerant to any mutant protein produced (23–25).

**Table 2:** FAP antigens in vaccines under development.

Protein/Antigen	Function Related to Polyp Formation	Mechanism of Immunogenicity
APC mutation	Mutation disrupts the normal function of the APC protein resulting in uncontrolled cell growth and polyp formation	“Neo-antigen”; the generation of cytotoxic T-cells against the mutation
ERB3	Erb-b2 receptor tyrosine kinase 3, is a receptor tyrosine kinase in the epidermal growth factor family whose kinase activity contributes to the formation of intestinal adenomas	Peptide based vaccines directed to extracellular domains given with antibody inducing adjuvants could generate antibodies blocking kinase signaling
Ascl2	Regulates intestinal stem cell fate and is highly upregulated in high risk polyps and colorectal cancer	Marked overexpression in polyps increases immunogenicity by unmasking cryptic epitopes
CDC25B	Cell cycle regulator that can be highly expressed in high-risk polyps that may develop into carcinomas	Marked overexpression in polyps increases immunogenicity by unmasking cryptic epitopes
COX2	Cyclooxygenase 2 is an enzyme responsible for producing prostaglandins which mediate inflammation	Marked overexpression in polyps increases immunogenicity by unmasking cryptic epitopes
EGFR	Epidermal growth factor receptor signaling stimulates the development of polyps in the intestines	Marked overexpression in polyps increases immunogenicity by unmasking cryptic epitopes

The second broad category of specific antigens expressed in the polyps of FAP patients are non-mutated aberrantly expressed tumor associated proteins. Several groups have studied vaccines using a single antigen to immunize the APC^Min^ in an attempt to inhibit or prevent polyp formation. In one study, investigators immunized mice with peptides directed against the oncoprotein ERB3 (26) (**Table 2**). Previous studies had shown that ERB3 was a dominant tyrosine kinase pathway in the APC^Min^ and signaling through ERB3 was a requirement for polyp formation (27). The investigators designed epitopes directed against extracellular domains that would generate endogenous antibodies which would bind to those domains and block ERB3 signaling. Similar endogenous antibodies have been shown to be generated to the HER2 protein after immunization. Vaccination of patients with HER2 peptides resulted in some individuals developing antibodies specific for a residue critical for HER2 signaling through an extracellular regulated kinase (28). The antibodies could suppress not only phosphorylation of HER2 in HER2 expressing cancer cells but inhibited colony formation in soft agar. Similarly, investigators immunized the APC^Min^ with the ERB3 peptides using KLH as an antibody inducing adjuvant (26). Immunization resulted in significantly fewer polyps in experimental vs. control mice and cell depletion studies demonstrated the anti-polyp effect was mediated through CD4 T-cells. Antibodies, but not T-cells, can cross the placenta and further experiments revealed that the offspring of the ERB3 peptide-immunized mothers had significantly less polyp formation as compared to the offspring of control mothers inferring the transfer of protective immunity from parent to offspring. An additional antigen that has been used for immunization in FAP is the achaete-scute family bHLH transcription factor 2 (Ascl2) (**Table 2**). Ascl2 is involved in Wnt signaling and is a master regulator of the intestinal stem cell (29). Ascl2 is highly overexpressed in polyps and the higher the expression of the protein the greater the likelihood of malignant transformation (30). Marked overexpression of self-tumor antigens has been shown to be a dominant mechanism by which immunity is enhanced (31). Overexpression unmasks subdominant epitopes which have not been available in great enough concentrations in the MHC for immune recognition (32). Investigators developed a novel vaccine platform designed to enhance the generation of immunity to a non-mutated antigen such as Ascl2. They created a chimeric protein consisting of the tumor antigen and a cell penetrating peptide aimed to improve antigen delivery into the innate immune cell and, thus, epitope presentation. The adjuvant included in the system consisted of a TLR2/4 agonist. The vaccine generated both antibody and T-cells specific for the antigen but was only effective at inhibiting formation of polyps as compared to control when given in combination with an immune checkpoint inhibitor, anti-PD-1 (29). Indeed, the addition of anti-PD-1 increased the level of polyp infiltrating CD3 T-cells approximately 2.5-fold over vaccine alone. The combination approach was well tolerated and could have promising activity in patients at the highest risk for polyposis progressing to invasive carcinoma.

Single antigen vaccines, such as those described above, have shown activity in a variety of murine cancer prevention studies but many investigations have demonstrated that multiple antigen vaccines are superior to immunizing with single antigens for cancer interception and prevention (33, 34). Multiple antigen vaccines can elicit a greater repertoire of T-cells trafficking to the tumor. Targeting a number of proteins offers a better chance of immune recognition of more antigens expressed in the lesion and with T-cells of multiple specificities very high magnitude lesion specific immune responses can be generated as compared to single antigen responses alone (33). Many non-mutated immunogenic proteins are expressed in intestinal polyps (17). Investigators evaluated gene expression in over 400 colorectal cancer and 50 adenoma gene data sets and identified genes that were highly upregulated (>2 fold) in both the adenoma and cancer but not in adjacent normal colonic tissue with the presumption that upregulated genes would potentially encode overexpressed proteins. One hundred and sixty genes were identified that were common to both. Using siRNA screens to silence those genes in a panel of colon cancer cell lines, the researchers prioritized those upregulated genes that appeared to have important functional effects on the growth and survival of the cancer cells. Twenty-three of those genes, when silenced, resulted in significant decreases in proliferation or increases in apoptosis (17). To date, nearly all of those genes have been shown to be immunogenic in humans and several have shown activity as vaccine immunogens in APC^Min^ mice (35). As examples, CDC25B and COX2 are both proteins that are highly overexpressed in lesions associated with FAP (36). CDC25B is a cell cycle regulator and phosphatase that is overexpressed in high-risk polyps and adenomatous polyps that may develop into carcinomas (**Table 2**). COX2 is a cyclooxygenase enzyme that produces prostaglandins in the colon (**Table 2**). We found patients with colon cancer had high levels of antibodies to these two antigens as compared to volunteer age matched donors (p>0.05) (35). Peptide epitopes predicted to bind to human class II MHC were identified for each protein and T-cells specific for both the peptides and corresponding recombinant protein were generated from human lymphocytes validating these proteins as human antigens. Some of the peptides were highly homologous between mouse and humans. Using the homologous peptides as vaccines, and immunizing mice, animals developed both peptide and protein specific IFN-γ-secreting T-cell responses to CDC25B and COX2. As single antigen vaccines, APC^Min^ mice immunized with CDC25B or COX2 peptides developed fewer small bowel tumors as compared to controls (p=0.01 and p=0.02 respectively). The anti-polyp immunity was mediated by CD8 T-cells and the remaining polyps of vaccinated animals were highly infiltrated with CD8 T-cells (35). We developed a tri-antigen peptide-based vaccine consisting of class II epitopes derived from CDC25B, COX2, and EGFR all non-mutated proteins overexpressed in FAP lesions (37) (**Table 2**). The vaccine was given alone to the APC^Min^, or in combination with naproxen over 18 weeks. Animals receiving vaccine alone showed a 33% inhibition of polyp formation while naproxen alone showed 54% inhibition (p<0.0001) compared to adjuvant alone. Combination treatment (vaccine+naproxen) demonstrated significantly greater inhibition of polyps than either modality (p<0.001), 81% inhibition vs. adjuvant. Antigen specific T-cells could be detected at higher levels than control in both the vaccine alone (p=0.0001) and combination-treated animals (p<0.0001). The magnitude of the systemic antigen specific immune response, as detected in T-cells derived from the spleen, was significantly correlated with lower polyp counts (p=0.0014). Vaccines combined with other immune modulators such as PD-1 directed monoclonal antibodies or cyclooxygenase inhibitors may synergize the immunologic effect of each agent resulting in superior control of polyp formation as compared to single modality therapy.

## Immune function and immune environment in FAP

Prior to initiating any vaccine or chemo-immunoprevention study in FAP patients, one must consider the immune microenvironment and any immune alterations caused by APC mutations. The weak immunogenicity of the antigens expressed in FAP lesions may not be the only obstacle to establishing anti-polyp immunity in FAP. APC germline mutations are associated with changes in immune cell function that could adversely impact the generation of clinically effective immunity after vaccination (**Table 3**). APC regulates cytoskeleton and migration functions across a variety of cell types. Most of the potential immune defects ascribed to APC mutations have been delineated using the murine APC^Min^ model. There have been few studies using human immune cells derived from patients with FAP and data have been difficult to interpret due to the limited number of samples studied and the heterogeneity of immune responses between individuals. In very preliminary studies, using T-lymphocytes derived from seven patients with FAP and seven age and sex matched volunteer donors, investigators evaluated T-cell migration and adhesion functions and found both to be significantly impaired in FAP patients (38). In more recent and extensive experiments of immune cell number and function, researchers studied 12 FAP patients and 12 age-sex matched volunteer controls against a variety of external stimuli (39). After T-cell or peripheral blood stimulation, comprehensive multiplex methods were used to assess the number, proliferation, function, and character of the responding adaptive and innate immune cells between the two groups. Results from the study showed there were minimal, but consistent differences in the levels of T-cells, NK cells, and some cytokines that were produced in response to stimuli. None were significant. However, the capacity of T-cells to migrate in response to chemokines was significantly reduced in the individuals with FAP as compared to controls (39). While these data are provocative, they underscore the need to focus further study on human immune cell functional testing of FAP patients prior to the initiation of chemo-immunoprevention approaches. While there are significant murine data to support a number of potential immune defects, many of these have not been validated in patient samples.

**Table 3:** APC mutation induced immune defects and correction strategies.

Immune Defect	Approach
Impaired T-cell migration and adhesion	These functions are linked via appropriate activation via professional antigen presenting cells such as dendritic cells (DC) or the use of Toll like receptor agonists
Defective T-cell activation	
Defective T-cell effector function	
Induction of PD-L1 expression	Combination with COX2 inhibitor Combination with anti-PD-L1 monoclonal antibody Combination with anti PD-1 monoclonal antibody
Numerically fewer CD8 cells	Use of cytokines that promote the differentiation and expansion of CD8 T-cells such as IL-12, IL-2, IL-7, and IL-15

**Figure 1:**
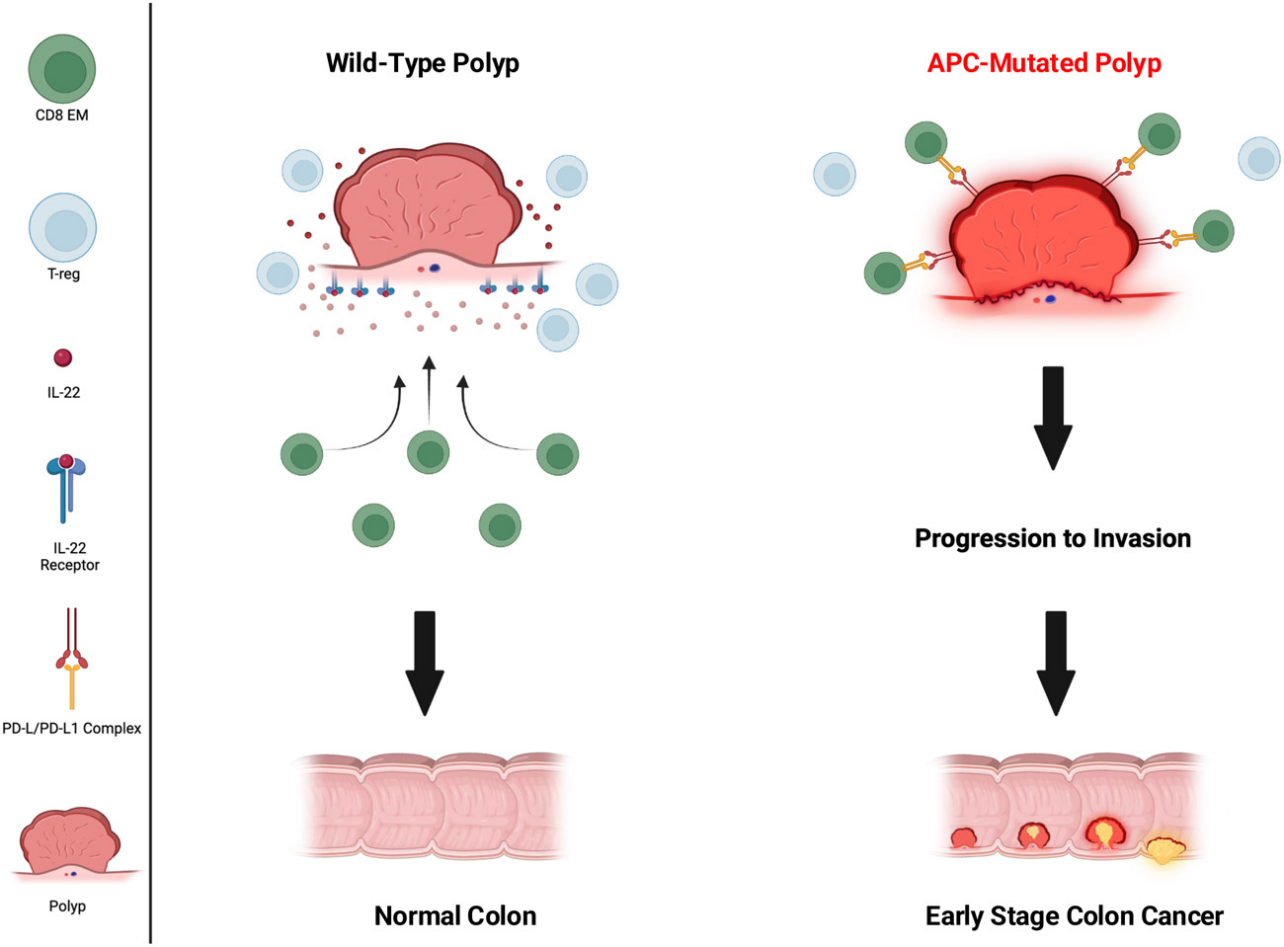
Immune environment of wild-type vs APC-mutated polyp. CD8 effector memory (CD8 EM) (green circles) active in wild type and inactivated in APC mutated, T-regulatory cells (Treg) (light blue circles) abundant in wild type, IL-22 (red dots), IL-22 receptor (blue receptor present only on wild type), PD-1/PD-L1 complex (yellow and red receptors present only on APC-mutated).

Murine models have unmasked several potential immune defects that may be related to germline APC mutations. APC plays a role in CD4 T-cell differentiation by controlling microtubule organization and the nuclear localization of Nuclear Factor of Activated T cells (NFAT). NFAT is a transcription factor needed for specific cytokine gene expression that drives T-cell differentiation. Investigators demonstrated that APC mutations reduced the presence of NFAT in the nucleus of intestinal Treg cells impairing their ability to differentiate (40). T-cells from APC^Min^ mice showed reduced cytokine production (IL-10) and a reduced Treg lineage commitment response (RORƔt) to TCR stimulation. Lack of functional Tregs in the intestine will result in increased inflammation and potentially significant changes in the gut microbiota due to development of low-level colitis. Additional studies have shown that intestinal epithelial cells with APC mutations are resistant to IL-22 due to reduced expression of IL-22 receptor and increased expression of STAT 3 inhibitors (41). IL-22 is a major cytokine operative in maintaining intestinal homeostasis and tissue regeneration (**Figure 1**). Such impaired responses further contribute to microbial dysbiosis. Indeed, the gut microbiota in FAP patients is different from volunteer donors and associated with a significantly decreased microbial richness (42). Investigators questioned how this limited microbiota might impact the development of the immune system and performed colonic biopsies on 14 volunteer donors and 13 patients with FAP to phenotype intraepithelial lymphocyte populations (43). Experiments found a loss of resident memory T-cells and Ɣδ T-cells in the tissues suggesting impaired cellular immunity. There is evidence that this *in vitro* observation has a real-world clinical correlate. A recent study evaluated 60 patients with APC mutations and 25 wild type individuals with colorectal cancer who received immune checkpoint therapy. In a multi-variate analysis, APC mutations predicted a worse response to immune therapy as compared to wild type (HR=4.25; 95%CI, 1.46-12.4, p=0.0008) suggesting impairment in the ability to mount an immune response (44). Patients with APC mutations also displayed less T-cell infiltration into their tumors as compared to patients without mutations which may suggest T-cell migration difficulties.

APC mutations may not only impact the function of cells of the adaptive immune system but also cells of the innate immune system as well. Investigators evaluated the role of APC deficiency in the function of DC by generating DC-specific APC knock-out mice. They found that loss of APC in DC resulted in fewer CD8 T-cells and more Tregs in tumors and draining lymph nodes in mice and attenuated anti-tumor immune responses (45). Moreover, the DC were less effective at priming CD8 T-cells and more preferentially induced Treg activity (**Figure 1**). The APC protein has functional domains that bind to β-catenin and other proteins to form a complex which targets β-catenin for degradation. Loss of APC allows β-catenin to translocate to the nucleus, bind to the PD-L1 promoter, leading to increased transcription of PD-L1 and its protein expression on cancer cells, thus, inhibiting tumor specific immunity (46). Presumably, this mechanism of increased expression of PD-L1 would be operative in other cells where APC mutations were present such as innate immune cells and polyps. This is yet another mechanism by which germline APC mutations, such as those seen in FAP, disrupt the ability to mount successful immune surveillance preventing the progression of polyps to colorectal cancer (**Figure 1**). Although further research needs to be done to fully understand the extent of immune impairment in patients with FAP, there are adjuvant approaches that can be added to vaccination that will improve the magnitude and function of the immune response generated against FAP associated antigens (**Table 3**).

## Considerations for the clinical translation of FAP vaccines

Will the potential immune defects attributed to APC gene mutations be a limiting factor for FAP vaccines and immunogenicity? Human clinical trials with detailed immune monitoring will give us answers. The vaccine studies described above, however, showed synergy between the administration of vaccines and other immune modulator combinations such as anti-PD-1 or naproxen which can down regulate PD-L1 on polyps (29, 34, 37). It will be possible to override some of the potential deficits in the ability to mount an immune response with well-designed vaccine adjuvants or vaccine delivery technologies as we have observed in clinical studies of more classic cancer vaccines (**Table 3**).

A class of adjuvant that is ideally suited to providing a “danger” signal to weakly immunogenic “self” proteins are Toll Like Receptors (TLRs) (47). TLRs are pattern recognition receptors that sense pathogens or damage that occurs to cells. Once ligated they initiate a strong innate immune response that can trigger a vigorous adaptive T-cell response. Used as an adjuvant, TLRs have been very successful in boosting high levels of immunity to cancer antigens. Another class of adjuvant that has shown promise in enhancing weak immune responses is the use of cytokines (48). Either as soluble proteins or encoded into DNA or mRNA, IL-15, IL-7, IL-12 and IL-21 have been particularly effective at stimulating Type I T-cell responses in combination with GM-CSF. Furthermore, IL-12, IL-7, and IL-15 can stimulate the proliferation and survival of CD8 T-cells *in vivo* which may be a problem induced by APC germline mutations in patients with FAP. One method to highly activate T-cells is to provide professional antigen presenting cells in the form of DC vaccines that have already been generated ex vivo and loaded with antigen. There are many more novel vaccine adjuvants and adjuvant systems under development (18).

The manner in which the vaccine is delivered can greatly influence its immunogenicity. For example, virus like particles (VLPs) are nanostructures that resemble viruses (49). The particles are recognized by the immune system as a virus, but are not infectious and can be engineered to display other antigens to present to the immune system. VLPs provide the danger signal which activates innate immune cells and tolerized antigen can be recognized in an immunogenic fashion. Nucleic acid immunization is also another highly immunogenic method of antigen delivery and ideally suited to less immunogenic proteins (50). Nucleic acids have inherent immunogenic properties and can be adapted to encode not only the antigen, but any adjuvant. There has been an explosion in research of vaccine delivery technologies and adjuvants designed to enhance the magnitude of immunity generated and prolong immune responses (51). The development of vaccines directed against FAP lesions will benefit from these discoveries.

## Conclusions

FAP can serve as proof of principle that once we have insight into what drives pre-malignant polyps toward adenomas and ultimately colon cancer, we can strategize ways to mitigate and/or completely avoid such progression. Having determined the APC gene as a major mutational driver in FAP patients, resulting in 100% risk of developing colon cancer if left untreated, allowed us to identify patients at the highest risk of cancer. Vaccines for cancer interception in FAP, if successful, would have the potential to significantly reduce the morbidity and mortality related to this disease. With a limited number of immunizations and periodic boosters, patients may be able to achieve polyp control or prevention of dysplasia without the need for oral daily medication or even surgery. This would be the goal of successful vaccination.
